# Letter from Dr. Stroud of Terrell, Texas

**Published:** 1905-08

**Authors:** L. M. Stroud

**Affiliations:** New York


### Letter From Dr. Stroud of Terrell, Texas.

NEW YORK, July 10, 1905.

*Dr. F. E. Daniel, Editor Texas Medical Journal, Austin, Texas.*

DEAR DOCTOR: Find enclosed $5 bill for five years subscription. The "Red Back" was one of the first of the thousands of journals on the long table in the reading room of the Polyclinic to greet my eyes.

One is impressed here, as elsewhere, that after all it is the man and not so much the school, and that there are two elements of success necessary. The ability to generalize underlying principles and to master the minutest details of technique.

We have just witnessed an operation by one of the cleverest men in the world for the radical cure of double oblique inguinal hernia. The amphitheater was not any cleaner than the good housewife would keep a department down in Texas. Immediately surrounding the patient everything was surgically clean. The patient's belly was scrubbed with some good old soap and a tencent brush, which had just been boiled in water. Next the belly was scrubbed with a 1 to 1000 mercuric chloride solution with another ten-cent brush. The field of operation was surrounded by hot, wet towels wrung out of the same solution. The towels were such as a lady will hand you if you call for laundered towels, pillow slips or sheets at any home, or as she will get at the next neighbor's if her laundry is out.

This world renowned author in surgery gently pecked the skin up over the usual site for the operation, and incised it and the underlying fascia; then with a few snips of the scissors the sheath of the external oblique was brought into full view, placing his index finger in the external abdominal ring he invaginated it for an inch so as to demonstrate the canal, then with a gentle snip of the scissors the canal was opened and the underlying cord raised and held out of the way by a broad band of sterile gauze. Just at this time the patient coughed and brought the hernial sack up into the wound. He said we always have them to do this and winked the other eye. He was working so easy and fast, and with so little injury to the tissues that the cough looked like a beautiful part of technique to oblige the class. He then resterilized his hands and with his index finger freed the sack from adhesions, both extra and intra abdominal, then raised the sack, tied it off and then anchored it immediately outside the internal abdominal ring by passing his needle through the opposed tissues and fastening his ligature to a roll of gauze over the skin.

Up to this time he had touched his wound but twice with his hand—when he invaginated the external ring to demonstrate the canal, and when he ran his finger inside the hernial sack to free any existing adhesions. We are taught it is nearly impossible to sterilize the hand and never to touch a wound with the naked hand when it can be done as well with instruments, and to conserve the energies of the tissues by as little traumatism as possible. In this case no muscles had been cut, but were opened by dividing a few bands of fascia with scissors. The illio-hypogastric and illio-inguinal nerves, which are in plain view when the external oblique is exposed, were pushed aside with blunt scissors. The surgeon then dissected the fascia loose from the portion of the internal oblique, which he then could bring well up to Poupart's ligament without tension of his sutures. He then sewed the fascia well together with fine silk, not sewing the muscles.

Thus he had met the three indications; removed the hernial sack; restored the obliquity of the canal and sewed his fascia with silk in order to hold four or five weeks, which is the shortest time this tendon can repair itself as is the case with any like tissue in any part of the body.

Well, what was remarkable about this simple operation? I will tell you. The surgeon then turned over the case to his first assistant and attendants and went up town. His assistants then operated upon the other side. The first thing he did was to criticise his great teacher by saying, "I do not anchor the stump of the hernial sack outside, but drop it back."

He then proceeded to operate, having operated hundreds of times and seen his great teacher operate thousands of times, likely. He divided the tissues freely with the knife down to the hernial sack, using his fingers for retractors, and paid no attention to the nerves, tied the hernial sack, dropped it back without either anchoring it or freeing it from adhesions, closed his wound with catgut sutures and did a brilliant operation quicker than his master.

Now, what may be the difference in results to the patient? His teacher thought if the hernial sack was freed from adhesions and anchored in the internal abdominal ring it would not only reinforce this weak point by an adhesive plug and help to restore the obliquity of the canal, and by freeing the sack from adhesions prevented intra abdominal pressure from tugging at the stitches every time the diaphragm moves in breathing, and that it was better to sew the external fascia with fine silk as cutgut was liable to be absorbed in four days and loosen the main support of all these muscles and invite an immediate hernia. He did not cut the nerves supplying the underlying tissues for fear of a remote hernia from thinning of the muscles of the flank from trophic changes within the next year or two. This assistant had worked with his great teacher all these years and had not "taken the cue from him."

This was illustrated the other day by one of the professors, who took his surgical staff over to the bacteriologist to teach them how to sterilize their hands. Think of it, a surgeon taking his trained assistants over to teach them how to sterilize their hands, and not one of them could take a sterile impression of the hand except the professor. With all their eyes they could not catch his tricks in scrubbing. One of them got mad and quit. The touch had not been given him and the poor fellow was lacking in "the personal equation."

Some men are educated apes, and to use an over-worked phrase, their common sense is the X, or unknown quantity. But education is power and often only makes a doctor all the more dangerous. I talk with educated men of broad experience who are riding hobby horses. One believes he can cure any case of meningitis with diphtheritic antitoxin; another can cure puerperal septicemia with salicylate of soda; another can cure whooping cough with a tight belly-band, and one who will not let a sick baby nurse a healthy breast because it is bad in milk infection, etc.

The people are at the mercy of these educated fools, and it is all right for our State Board to eliminate bootblacks and cowboys from the profession. They should have a test for common sense. For instance, test the applicant's powers of observation and perception by bringing in some "sheep-sorrel" and see if he can tell it from parched coffee without tasting it.

Men come up here or go to Chicago or New Orleans and see a little surgery and tackle the first patient with colic, on their return, and get his appendix to put in their private museum. It has been suggested that this will be regulated by the survival of the fittest. This is a slow and cruel law. Some of the biggest asses I have known were perfectly sound in body and did not even carry life insurance. Have about concluded God put the people here for the doctors to experiment on, and what one fool can't think of another can.

Nature strikes the balance sheet at the grave yard. It seems to be Nature's scheme to get one generation out of the way of the next. If the chemist invents more germicides, God compensates by inspiring a few more damn fools with an "insane desire" to read medicine. It will all likely be the same in a hundred years. Nature makes few mistakes.

So the Irishman was right who said "one man is just as good as another and as to that a great deal better."

L. M. Stroud.

